# Postoperative hemorrhage after Le Fort I osteotomy hemostasis with angiographic embolization: report of two cases

**DOI:** 10.1093/jscr/rjad663

**Published:** 2023-12-14

**Authors:** Yasutaka Yun, Shiro Kurasawa, Mizuki Morita, Risaki Kawachi, Hideyuki Murata, Shunsuke Sawada, Yuka Kojima, Masao Yagi, Mikiya Asako, Hiroshi Iwai

**Affiliations:** Department of Otorhinolaryngology, Head & Neck Surgery, Kansai Medical University, Hirakata, Osaka, Japan; Department of Otorhinolaryngology, Head & Neck Surgery, Kansai Medical University, Hirakata, Osaka, Japan; Department of Otorhinolaryngology, Head & Neck Surgery, Kansai Medical University, Hirakata, Osaka, Japan; Department of Otorhinolaryngology, Head & Neck Surgery, Kansai Medical University, Hirakata, Osaka, Japan; Department of Otorhinolaryngology, Head & Neck Surgery, Kansai Medical University, Hirakata, Osaka, Japan; Department of Oral and Maxillofacial Surgery, Kansai Medical University, Hirakata, Osaka, Japan; Department of Oral and Maxillofacial Surgery, Kansai Medical University, Hirakata, Osaka, Japan; Department of Otorhinolaryngology, Head & Neck Surgery, Kansai Medical University, Hirakata, Osaka, Japan; Department of Otorhinolaryngology, Head & Neck Surgery, Kansai Medical University, Hirakata, Osaka, Japan; Department of Otorhinolaryngology, Head & Neck Surgery, Kansai Medical University, Hirakata, Osaka, Japan

**Keywords:** Le Fort I osteotomy, postoperative complication, epistaxis, descending palatine artery, angiographic embolization

## Abstract

This study reported two cases of acute life-threatening hemorrhage after Le Fort I osteotomy. In both cases, computed tomography and angiography revealed damage to the descending palatine artery, which was successfully treated by angiographic embolization. Although massive hemorrhage after Le Fort I osteotomy is rare, acute hemorrhage from the postoperative area may occur. Angiographic embolization is useful in cases of such hemorrhage from the posterior nasal cavity where endoscopic hemostasis is not possible.

## Introduction

The Le Fort I osteotomy is a common maxillofacial surgery [1]. This method is used to fix many malocclusions and maxillofacial abnormalities. In this procedure, the pterygoid plates are separated by cutting at the junction of the plates from the maxilla [1]. Postoperative complications are rare; however, serious epistaxis has been reported [2, 3].

Epistaxis is caused by the separation of the pterygoid fragment from the maxilla, which damages the blood vessels running inside the bone [1]. The internal maxillary artery and its terminal branches are vessels involved in such massive hemorrhage, and the descending palatine artery is the common source of massive hemorrhage [4, 5].

Hemostasis from the vessels deep in the nasal cavity, such as the descending palatine artery, frequently cannot be performed using common hemostatic methods of topical vasoconstriction, nasal packing, and cauterization [6]. Furthermore, the bleeding point may often not be visualized for hemostasis by endoscopy in the postoperative nasal cavity [6]. Thus, angiographic embolization techniques provide the most effective hemostasis [7].

Herein, we report two cases of postoperative hemorrhage from the descending palatine artery, a terminal branch of the internal maxillary artery, which was treated with angiographic embolization.

## Case report

### Case 1

**Figure 1:**
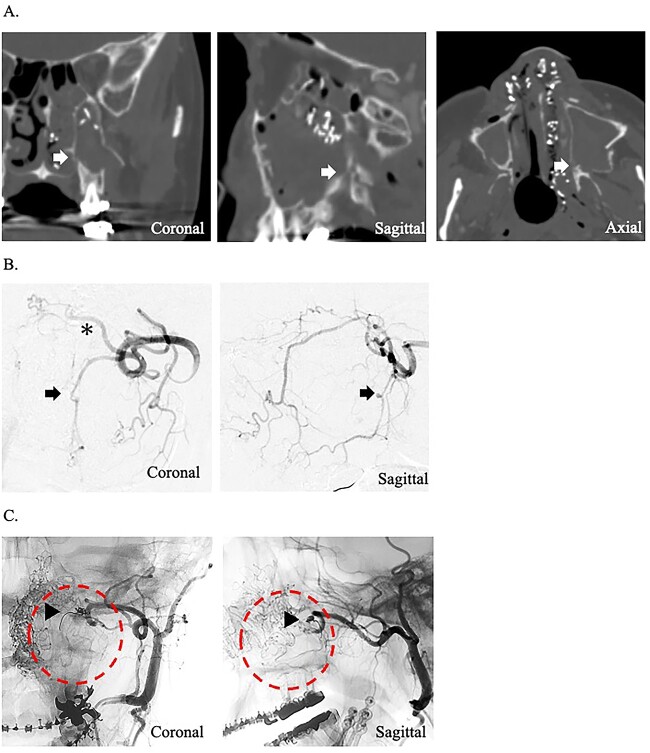
(A) Postoperative coronal, sagittal, and axial computed tomography images, which show the separated bone parts of the left maxilla (arrow). (B) Coronal and sagittal selective angiographic images from the left internal maxillary artery. The arrow indicated a pseudoaneurysm of the left descending palatine artery. The asterisk indicated the left sphenopalatine artery. (C) After embolization and coronal and sagittal selective angiographic images. The Triangular arrow indicated the endovascular coils, and the circle indicated the disappearance of the vascular flow area.

A 49-year-old woman with severe hemorrhage after Le Fort I maxillary osteotomy and sagittal split ramus osteotomy was transferred to our hospital. She underwent surgery for a jaw deformity 7 days earlier by the dentist at another hospital, and postoperative Day 5, severe epistaxis from the left nasal cavity occurred. At the previous hospital, the patient was treated for epistaxis by nasal tampons; however, the hemorrhage could not be completely stopped. After transport, an endoscope was used to search for the bleeding point from the left nasal cavity. The source of hemorrhage was identified near the natural ostium of the maxillary sinus. This massive was impossible to control under endoscopy; therefore, temporary hemostasis was used with nasal packing. Contrast-enhanced computed tomography of the paranasal area revealed that the maxilla and the pterygoid fragment were cut and separated. A cut of the bone was also found around the descending palatine artery, and blood was found within the maxillary sinus (Fig. 1A). The preoperative hemoglobin concentration was 12.6 g/dL, and the hematocrit was 38.0%. After transport, hemoglobin concentration slightly decreased to 11.2 g/dL and hematocrit to 34.6%. Since the bleeding point could not be identified by endoscopy and the hemorrhage continued, an angiographic embolization by a radiologist was selected as the hemostatic method. The angiography showed a pseudoaneurysm of the left descending palatine artery, on which embolization was performed by endovascular coils and gelatin sponges (Fig. 1B and C). Although hemostasis was achieved adequately after angiographic embolization, the hemoglobin concentration and hematocrit were further decreased to 9.8 g/dL and 30.0%, respectively.

### Case 2

**Figure 2:**
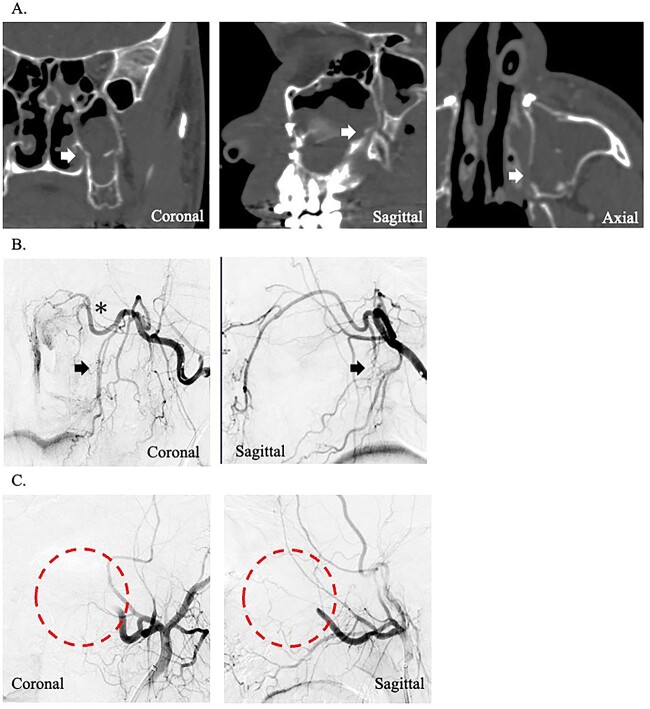
(A) Postoperative coronal, sagittal, and axial computed tomography images, which show the separated bone parts of the left maxilla (arrow). (B) Coronal and sagittal selective angiographic images from the left internal maxillary artery. The arrows indicate the suspected region of injury to the left descending palatine artery. The asterisk indicated the left sphenopalatine artery. (C) After embolization and coronal and sagittal selective angiographic images. The circle indicated the disappearance of the vascular flow area.

A 24-year-old man presented to our department with massive epistaxis from the left nose. The patient underwent Le Fort I maxillary osteotomy and sagittal split ramus osteotomy for jaw deformity 10 days earlier by the dentist at another hospital and had repeated epistaxis episodes after hospital discharge. After the patient came to our hospital, the left nasal cavity was examined endoscopically; however, the hemorrhage had stopped, and no definite bleeding points were identified. Contrast-enhanced computed tomography of the paranasal area revealed that the maxilla and pterygoid fragment were cut and separated. In addition, a cut of the bone was found around the descending palatine artery, as in case 1 (Fig. 2A). The preoperative hemoglobin concentration was 14.2 g/dL, and the hematocrit was 40.0%. After hemorrhage, the hemoglobin concentration (11.3 g/dL) and hematocrit (32.4%) decreased. After hospitalization, massive hemorrhage recurred, and hemostasis was impossible by endoscopy. Therefore, angiographic embolization by a radiologist was selected. The angiography revealed vascular injury to the left descending palatine artery, and embolization was performed by gelatin sponges (Fig. 2B and C). As in case 1, hemostasis was sufficiently complete; however, after angiographic embolization, the hemoglobin concentration and hematocrit severely decreased to 9.3 g/dL and 27.5%, respectively.

## Discussion

Le Fort I osteotomy is commonly used for the correction of malocclusion and maxillomandibular deformities [1], which allows movement anteriorly/posteriorly, vertically, rotationally, and with segmentation and expansion [8]. The procedure was named in reference to the horizontal fracture pattern named Le Fort I, published by Rene Le Fort in 1901 [9].

Le Fort I osteotomy is initiated at the lateral maxillary buttress and extended through the piriform rim using a reciprocating saw. The same osteotomy is performed on the contralateral side. A thin osteotome is used to complete the posterior osteotomies of the lateral and medial maxillary buttresses. The nasal septum is separated from the maxilla using an osteotome. The posterior maxillary wall is then fractured with an osteotome. The osteotomies are completed, and a down fracture was made [1, 8].

Le Fort I osteotomy is generally very safe and has a low complication rate, ranging from 4 to 9% [10–13]. Of those, the incidence of postoperative hemorrhage which usually occurs within 10 days postoperatively is reported to be low (0%2%) [2, 10]. Given the few reported cases of such serious complications, optimal treatment guidelines for complications have not been fully established.

In cases 1 and 2, hemorrhage appeared within 10 days postoperatively, and hemorrhage from the descending palatine artery was suspected. In case 1, a pseudoaneurysm was detected.

Bykowski *et al.* reported that hemorrhage after a Le Fort I procedure occurs on average 10.6 days later [4]. The most common causative vessels are the maxillary artery, descending palatine artery, and sphenopalatine artery; they are injured when the maxillary tuberosity is separated from the pterygoid plates with an osteotome or during a down fracture [4].

The descending palatine artery is in the greater palatine canal in the vertical portion of the palatine bone. This artery is a peripheral branch of the maxillary artery that branches and remains the major artery that supplies the hard palate [1, 5]. This artery was reported to be easily injured during osteotomy of the medial or lateral maxillary sinus walls or during down fracture of the maxilla [5] and was reported as a causative vessel in 16.7% [2, 4].

Hemorrhage occurring immediately after surgery suggests a sharp injury to the vessel, whereas hemorrhage occurring a few days after surgery suggests a pseudoaneurysm caused by blunt injury to the vessel. Pseudoaneurysms occur when a vessel wall is partially disrupted, resulting in a hematoma, which is wrapped by the vascular adventitia or perivascular soft tissue. Therefore, pseudoaneurysms have less support from the vessel wall and are at high risk of rupture [14]. Nasal hemorrhage from a pseudoaneurysm was reported to be severe and requires emergency hemostasis [4].

The lifetime incidence of epistaxis is 60% in the general population, with hemorrhages from Kiesselbach’s plexus (anterior inferior septum in Little’s area) accounting for ~90% of the total [6, 15, 16]. This hemorrhage is from the anterior vessels and is usually not serious, and hemostasis can be accomplished with a combination of topical vasoconstriction, nasal packing, and cauterization [6]. However, posterior epistaxis is rare (10%), and the source of posterior epistaxis is the internal maxillary artery branch, which is often more severe than anterior epistaxis [6]. Identification of the bleeding point is important for hemostasis, and hemostasis is usually achieved using a nasal endoscope.

The success rate of hemostasis by endoscopy ranges from 80 to 94% [17]. Furthermore, an alternative approach for patients with difficult-to-control hemorrhage is angiographic embolization [7, 18–20]. Angiographic embolization of the distal branch of the maxillary artery is a useful technique in the management of hemorrhage following maxillofacial trauma or orthognathic surgery [21].

Embolization is performed with intravenous sedation and local anesthesia. Catheterization is performed via the femoral artery, with angiography of the external carotid artery to the internal maxillary artery. The angiographic catheter is approached through the origin of the external carotid artery to embolize the main branches of the internal maxillary artery, specifically the sphenoid and descending palatine arteries, where hemorrhage was suspected. Gelatin sponges in a dilute solution of iodine-based contrast medium are used for embolization [22]. The success rate of this procedure ranges from 87 to 93%, comparable with that of hemostasis by endoscopy [23].

In cases 1 and 2, the vascular injury caused by Le Fort I osteotomy was in a deep area that could not be examined by endoscopy, and a massive hemorrhage occurred. Therefore, angiographic embolization was selected for immediate hemostasis, and it was successful.

## Conclusion

We treated two cases of hemorrhage after Le Fort I osteotomy by angiographic embolization. This procedure was useful in cases of massive postoperative hemorrhage that could not be controlled by endoscopy.

## Data Availability

All data required are mentioned in the manuscript.
